# The prototype of a frailty learning health system: The HARMONY Model

**DOI:** 10.1002/lrh2.10401

**Published:** 2023-11-23

**Authors:** Kirsten J. Parker, Louise D. Hickman, Julee McDonagh, Richard I. Lindley, Caleb Ferguson

**Affiliations:** ^1^ School of Nursing, Faculty of Science, Medicine & Health University of Wollongong Wollongong New South Wales Australia; ^2^ Centre for Chronic and Complex Care Research Blacktown Hospital Western Sydney Local Health District Blacktown New South Wales Australia; ^3^ University of Wollongong Wollongong New South Wales Australia; ^4^ Westmead Applied Research Centre University of Sydney Westmead New South Wales Australia

**Keywords:** consumer engagement, frailty, implementation science, learning health system, model of care

## Abstract

**Introduction:**

Rapid translation of research findings into clinical practice through innovation is critical to improve health systems and patient outcomes. Access to efficient systems of learning underpinned with real‐time data are the future of healthcare. This type of health system will decrease unwarranted clinical variation, accelerate rapid evidence translation, and improve overall healthcare quality.

**Methods:**

This paper aims to describe The HARMONY model (acHieving dAta‐dRiven quality iMprovement to enhance frailty Outcomes using a learNing health sYstem), a new frailty learning health system model of implementation science and practice improvement. The HARMONY model provides a prototype for clinical quality registry infrastructure and partnership within health care.

**Results:**

The HARMONY model was applied to the Western Sydney Clinical Frailty Registry as the prototype exemplar. The model networks longitudinal frailty data into an accessible and useable format for learning. Creating local capability that networks current data infrastructures to translate and improve quality of care in real‐time.

**Conclusion:**

This prototype provides a model of registry data feedback and quality improvement processes in an inpatient aged care and rehabilitation hospital setting to help reduce clinical variation, enhance research translation capacity, and improve care quality.

## 1 BACKGROUND

Research waste is estimated at a cumulative 85% of research investment, thus costing billions of dollars worldwide across different clinical conditions.^1^, ^2^, ^3^ Whilst innovation and change are constant, performance has flatlined. Braithwaite et al. 2020 describe health as a paradox, with only 60% of care as evidence‐based, 30% as low value and 10% as harmful.^4^ For three decades this has gone unchanged. Knowledge translation, being the process of research learnings applied to practice, is used in healthcare to produce improvements in clinical care.^5^, ^6^ However, the translational continuum,^7^ which involves the application of research findings into practice, is currently estimated to take up to 17 years with widely variable uptake.^5^, ^8^ Research collaboration with clinicians and front‐line health staff, who are ultimately responsible for research adoption, is essential to timely knowledge translation.^9^, ^10^ Co‐creation and implementation alongside clinicians across the entire research trajectory will provide opportunities for rapid tangible health impacts.^9^, ^10^ The long‐standing historical time delay in implementation confirms that translation of evidence‐based research is challenging and multifactorial, but when prioritised will reduce the evidence‐practice gap, advance the quality of care, and enhance patient outcomes.^11^

Impactful real‐time use of routinely collected big data (eg, electronic health records or clinical quality registries) can create an accurate picture of the health status of populations.^11^ Big data is defined as vast complex data sets with the potential for multiple data sources which are widely variable, high in volume and increasing in velocity and size.^12^ There is a growing need for big data and its IT infrastructures, such as visual dashboards, to support the progression of practice improvement and promote accessibility and usability to end‐users. The generation of knowledge processes embedded into routine practice is the core strategic goal of a learning health systems model.^13^ Increasing understanding and appreciation of this model offers a platform to generate and formulate real time data‐driven evidence in a symbiotic relationship and the translation of research into clinical practice.^14^, ^15^

### 1.1 Learning health system models

A learning health system model prioritises knowledge acquisition and translation between research, healthcare, and clinical settings to improve quality of care and patient outcomes. A learning health system model was developed by the Institute of Medicine's (IoM) Roundtable on evidence‐based medicine in 2006^16^ and at its origin defined the learning health system as a dynamic model of care, which is a multifactorial and adaptable platform for learning that can constantly develop.^13^, ^17^ The creation of health systems and up‐to‐date data science platforms to support quality and decision‐making while in the clinical setting is a core component of the learning health systems model.^14^ This organised system of timely data learning amplifies the benefits through active participation and engagement of clinicians and stakeholders to support care decisions.^13^ Historically, clinical care and research have been somewhat considered separate entities with independent modes of priorities, and the model challenges this dichotomy.^17^ This strength of the learning health system model means it continues to capture ongoing individual priorities, accelerators, and challenges rather than a single‐point in time snapshot of care. The cyclical processes of the learning health systems model sees the translation of data to knowledge, knowledge to practice and then practice to data.^18^ Challenges remain as the model relies heavily on access to up‐to‐date clinical developments, clinician and stakeholder engagement and clinical leadership, which are all extrinsically impacted.^19^ Successful implementation sees developed processes that promote a cohesive and communicative system that works to achieve a shared strategic goal, and leverage research knowledge translation for point‐of‐care improvements for patients.

### 1.2 Clinical quality registries

Clinical quality registries incorporate a health‐specific database that pertains to a particular cohort of choice and can be used to underpin research, improve quality of care and provide information on the chosen data collection items.^20^ Clinical quality registries systematically collect data items, collates, and provides feedback on this information for healthcare stakeholders. These registries are governed by specific operational, governance and technical requirements.^21^ The use of clinical quality registries for data collection and research has been broadly utilised worldwide and originated as epidemiological data to measure population trends and overall health.^22^ Key advantages include their ability to provide benchmarks and performance data about practice standards and quality of care. Facilitating feedback on the variables they collect in a combined database of trends and patterns in treatments or interventions.^23^ Common criticisms of clinical quality registries include the data‐delay feedback and the poor integration of these data platforms with ever‐developing medical record systems (e.g. Electronic Medical Records). Large variability in registry methodology has brought about the importance of creating quality registries, and hence the current significant focus on improving ‘registry science’. Ultimately, registries are surveillance‐based research and observe patients throughout a timeline without directly adjusting and altering patients' care in any way.^24^

### 1.3 The role of implementation science

Implementation science includes different principles of the application of knowledge and skill development. It is the study of factors that impact the complete and effective use of innovations in clinical practice. It guides researchers and clinicians on the application and implementation of findings to improve care standards.^25^ Implementation science has several different frameworks to conceptualise their workings, examples include COM‐B (Behaviour Change Wheel in the context of Capability, Opportunity and Motivation),^26^ the RE‐AIM (Reach, Effectiveness, Adoption, Implementation, Maintenance)^27^ and PARIHS (Promoting Action on Research Implementation in Health Services),^28^ which have been synthesised from the barriers and enablers of research implementation.^29^ These theoretical frameworks aim to successfully integrate research knowledge into evidence‐based practice, which is a shared goal of the proposed HARMONY Model. Understanding various methods of implementation science help to recognise the operationalisation of the HARMONY Model. Ultimately through successful implementation science, practice reflects patient needs and improves the delivery of various health services.^30^ In the current Australian healthcare system, there are data‐based systems that are intuitive and capable of providing feedback through interactive displays of patient information, however, these need to be better formalised.

## 2 RESEARCH QUESTION

How to integrate big data in real‐time to develop a frailty learning health system prototype which improves care and identifies people most at risk of adverse outcomes?

## 3 AIM

To integrate big data in real‐time to develop a frailty learning health system prototype that improves care and identifies people most at risk of adverse outcomes by leveraging big data analytics in healthcare.

This paper describes a frailty learning health system prototype that integrates the distribution of real‐time data from primary data sources into a system of care that prioritises improved patient outcomes; namely the HARMONY Model (acHieving dAta‐dRiven quality iMprovement to enhance frailty Outcomes using a learNing health sYstem). The model provides a mechanism that leverages primary data sources to transform them into usable and practical formats. This paper uses Electronic Medical Records (EMR) and the Western Sydney Clinical Frailty Registry as the case study example. Transforming the use of this data can help to identify areas for priority practice improvement and informed decision making on local initiatives and future research direction. Foundations of this prototype are based on important concepts of implementation science^31^ and behaviour change^32^ to engage and align the goals of clinicians, researchers, key stakeholders, patients and consumers.

## 4 THE WESTERN SYDNEY CLINICAL FRAILTY REGISTRY

The Western Sydney Clinical Frailty Registry (WSCFR) is a dual‐site clinical registry that was developed through a collaboration between the University of Wollongong/Western Sydney Local Health District (WSLHD) Centre for Chronic and Complex Care Research, Westmead Applied Research Centre, University of Sydney and the Rehabilitation and Aged Care Service (RACS) and, Blacktown & Mount Druitt Hospitals within WSLHD. This prospective clinical cohort study set out to explore the condition of frailty and management within the community. This study aims to establish a clinical profile of frail patients over 5 years. These participants are screened daily by a Frailty Research Clinical Nurse Specialist for eligibility for the registry before a formal consent procedure. Eligible patients for inclusion were those admitted to either Blacktown or Mount Druitt Hospital under Rehabilitation and Aged Care Services (Geriatrics) and, aged 65 years and older. Inclusion is not limited by clinical condition and if cognitive impairment prohibits consent, then a next‐of‐kin is utilised. Those excluded were non‐English speaking, those under formal legal guardianship, not a resident of Australia or those whose follow‐up may not be possible.

The clinical registry follows patients along a timeline incorporating frailty status, associated multi‐morbidity and outcomes including driving status, institutionalisation, hospitalisation, and mortality status. The clinical registry incorporates a baseline assessment completed with the patient (Patient Reported Outcome Measures and Patient Reported Experience Measures) and collation of online EMR data including medications and some select blood results. Participants are then contacted by phone, at the 3‐, 6‐ and 12‐month time points to complete a verbal survey that investigates their readmission, health status and current driving status. The study has also been designed for future tracking of participant healthcare use at 1, 2, and 5 years through access to Medicare, Death Registry and Pharmaceutical Benefits Scheme.

The Clinical Frailty Registry was established in 2020 and is registered on the Australian Register of Clinical Registries (ACSQHC‐ARCR‐095).^33^ Currently, feedback from the registry to clinicians is delivered at departmental meetings, as described in Table 1. Further details of the Registry methodology can be read in the study protocol published in *Collegian*.^34^

**TABLE 1:** Case study snapshot of current practice.

Case study
*Blacktown & Mount Druitt Rehab & Aged Care Department* Current periodic analysis and feedback to the multidisciplinary team occurs at regular departmental meetings whereby Doctors, Nurses and Allied Health staff can view current health status of participants including frailty score, medications, mortality and rehospitalisation over 12 mo. At a recent departmental meeting looking at the current outputs and priority focussed areas, visualisation and feedback of prescribed medications during admission was discussed. Presented in a retrospective rudimentary dashboard which helped to facilitate discussion around medication usage and polypharmacy within study participants. This prompted reflections on medication prescribing and treatment practices amongst health staff.

## 5 THE FRAILTY LEARNING HEALTH SYSTEMS PROTOTYPE: THE HARMONY MODEL

Implementing what we learn from the Western Sydney Clinical Frailty Registry and EMR, utilising a learning health systems model and drawing upon implementation science methods, a new prototype for frailty knowledge acquisition and translation is proposed. The Frailty Learning Health Systems prototype (The HARMONY Model) aims to network frailty longitudinal data into an accessible and useable format for learning and practice improvement.

### 5.1 Rationale

**FIGURE 1:**
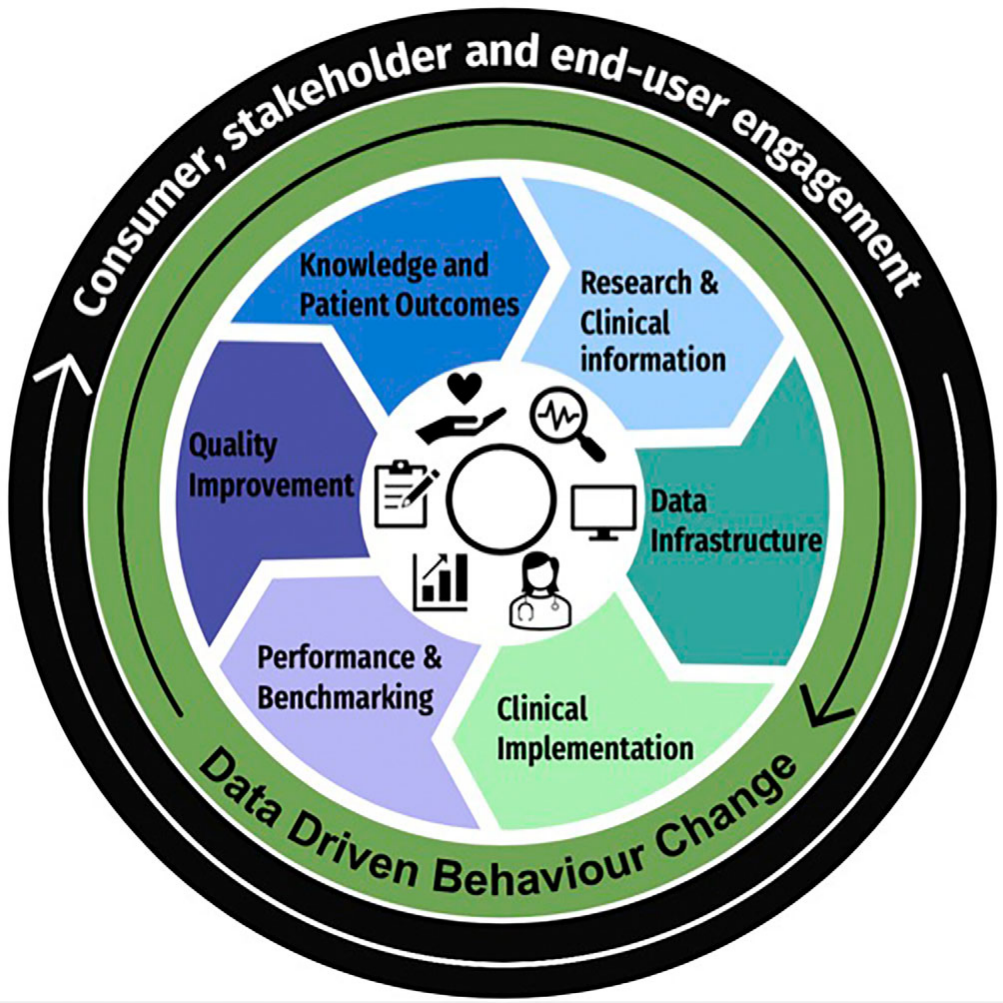
The frailty learning health systems prototype: The HARMONY Model.

The HARMONY Model aims to advance the quality of care for frail older adults admitted to hospital through timely data availability and shared decision making on health priorities. Inpatient hospital stays can have negative impacts on overall health and can lead to increased readmissions, mortality and reduced time spent at home. There is a growing need for the efficient use of big data, to have a purposeful role in practice development. This model is creating a local capability of data infrastructures to translate practice change into hospital settings to improve patient outcomes and overall quality of care. This model presents a way in which to network current data infrastructures for the benefits of patients, families, clinicians, hospitals and communities (as demonstrated in Figure 1). Creating better timely optics to understand how we can best meet patient needs and reduce risk factors associated with rehospitalisation and adverse outcomes.

### 5.2 Operational processes

Currently in the Australia there are intuitive systems that are data‐based, but often timely feedback is not feasible within operational constraints. As health systems advance and develop, the creation of a data dashboard can be implemented and utilised within local hospital settings. This prototype utilises a learning health systems model, registry data and EMR to demonstrate the potential of data infrastructures' effective and sustainable application in real‐time. Understanding that developments in machine learning are continuously occurring, moving to create an up‐to‐date platform that is versatile in its prioritisation of outcomes is necessary. This construct and model have the possibility to be adapted to various clinical settings and developed using multiple diverse data platforms that are catered to the specific clinical needs of patients or interests of clinicians.

**FIGURE 2:**
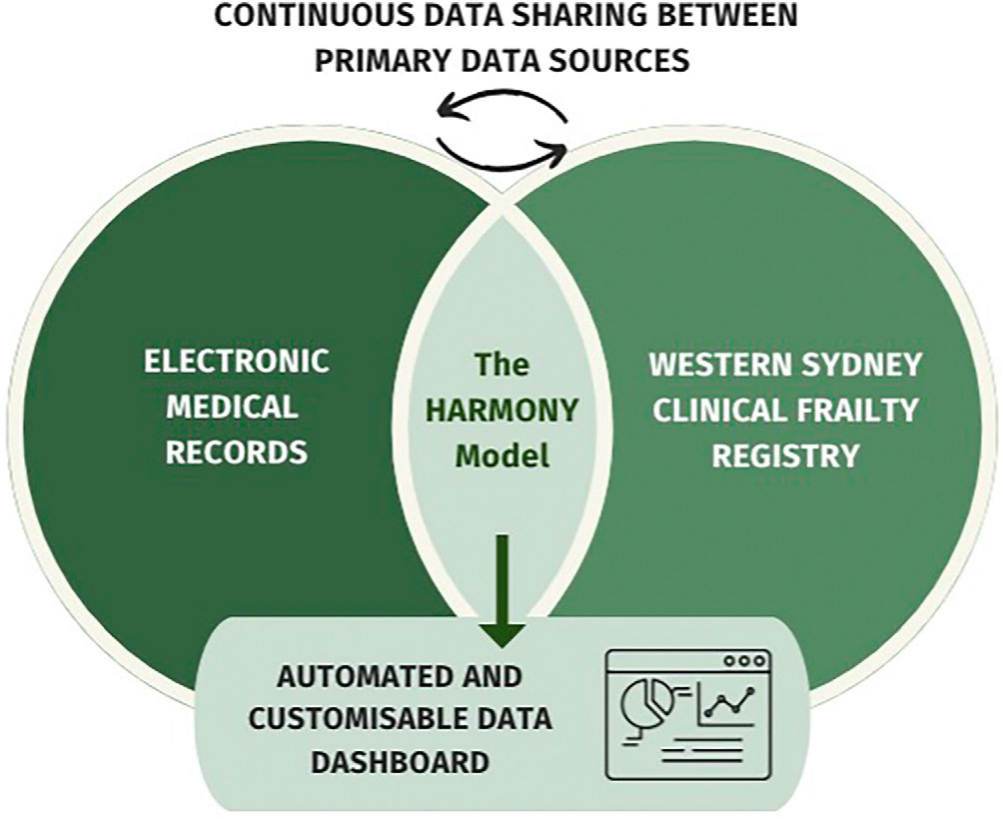
Operational processes and relationships of the HARMONY model.

The local‐level leveragability of a real‐time clinical picture of patients currently admitted to the aged care wards provides a clear direction for care provision and management. End‐user and consumer engagement throughout the data feedback processes ensures the continual prioritisation of patient centred care. As an example, clinicians can request specific representation of clinical data, and this can be implemented in the next model development cycle. The primary data sources have continuous data sharing capabilities, the current proposed layout of the HARMONY Model uses EMR and the registry as a case study example, but possibilities exist to adapt the model to incorporate more primary data sources. Continuous sharing and networking of varying data between sources is organised and collated by the HARMONY Model and produces automated outputs, visually represented in Figure 2. Therefore, the combination of this digital architecture and rapid research translation that the learning health system provides sees the possibility of transferable and measurable health impact, and overall improved patient outcomes.

### 5.3 Design and data dashboard

The HARMONY model will collate relevant frailty data on patients currently admitted and portray them in a data‐dashboard format for clinicians to utilise. The use of audit and feedback when used in combination with a data dashboard endeavours to combat challenges of accessibility and usability. This collaborative approach to learn as a community provides a multitude of possibilities to empower clinicians into dynamically improving the quality of care at a local level, which in time will help to improve the overall health and wellbeing of these populations. The micro‐level applicability of the model sees usual physical and cognitive assessments of frail older people on admission being utilised to formulate a clear picture of these populations and current clinical abilities. The model can also produce daily, weekly and monthly reports on patients' clinical conditions and other health related data to better understand trends and performance. This has clear capabilities to reduce unwarranted clinical variation, adverse events, and readmissions for these vulnerable populations.

**FIGURE 3:**
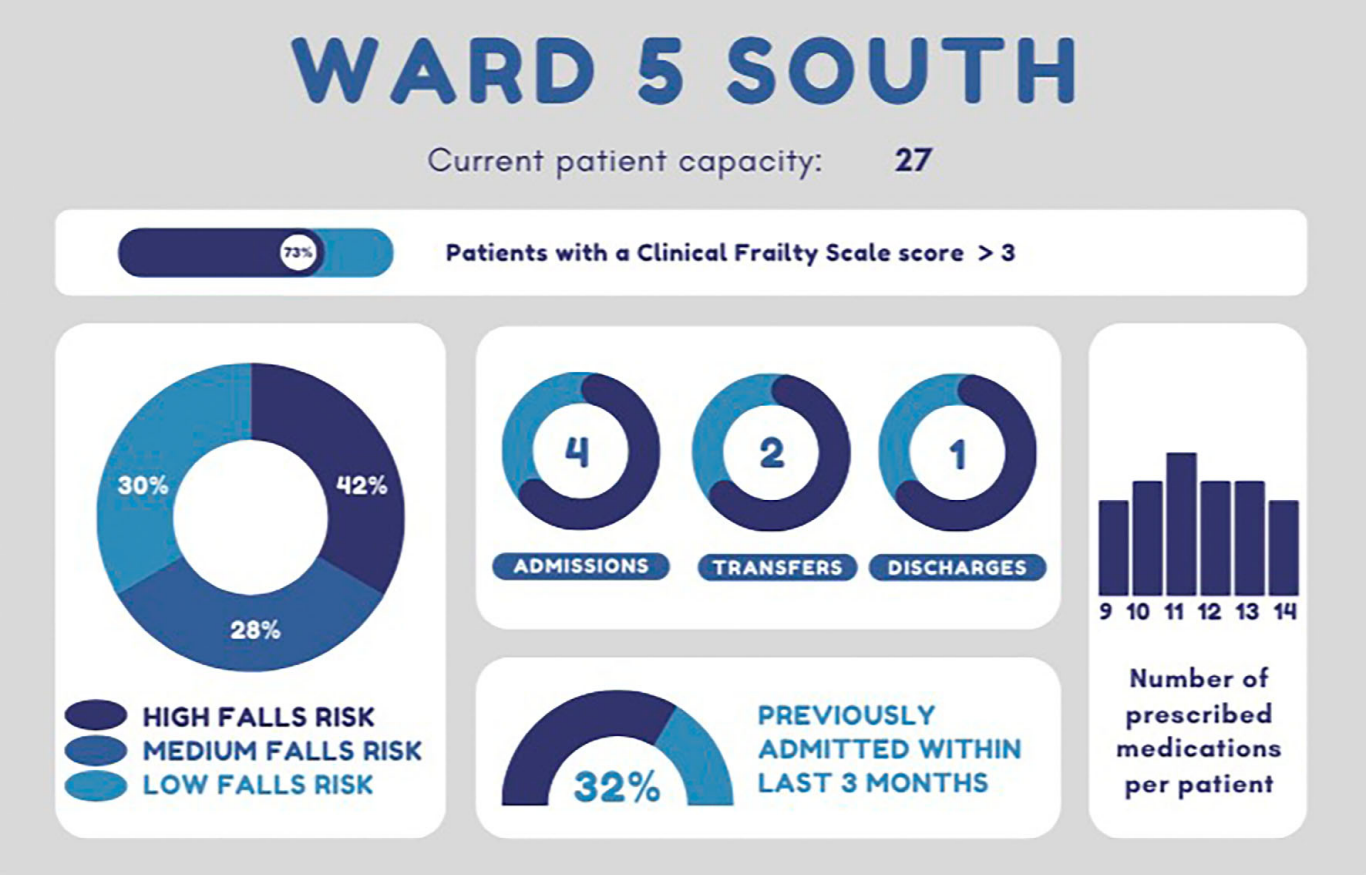
Proposed mock data dashboard example.

With the implementation of a simple visual configuration (via a dashboard) of trends of medications and readmissions, as two examples, clinicians can make accurate and relative decisions to dictate the organisation and prioritisation of care. As trends and analysis of data are visually represented, this can be utilised alongside multidisciplinary team grand rounds and journey board meetings where concerns for patient safety can be voiced. Furthermore, there are opportunities to incorporate data elements into clinical handovers to help prescription of future care direction and highlight patients of risks. These data help to consolidate and emphasise what clinicians already know about their patients in a system‐appropriate and coding equivalent manner. Using specific examples as demonstrated in Figure 3, a graph and statistics demonstrating that 32% of the patients currently admitted on the ward have been readmitted within the previous 3 months will help to prompt clinicians to better consider the transitional care needs of their patients. Or that over 70% of patients currently admitted are prescribed 10 medications or more, meaning on rounding Geriatricians can be aware and help to reduce polypharmacy in their patients.

## 6 STRENGTHS AND CHALLENGES

The HARMONY Model holds strength in the ability to develop knowledge generation processes around frailty to be embedded into routine practice and works to incorporate behaviour change strategies to create value‐based health care. Clinicians have an integral role in the development of the prototype and this will help to guide the elements and layout to meet the needs of those that will use it (themselves). Thereby creating supportive and collaborative environments for the design and production, which will in turn lead to successful implementation and application. The HARMONY model will help to facilitate better holistic and comprehensive inpatient hospital care by accessing current patient population data. Although this is the case, achieving timely reporting, where ‘real‐time’ is not possible is complex. Elements of data inaccuracies and freshness present limitations in the efficient translation of data to the dashboard. These challenges will affect implementation and ongoing perceived benefit, but as this model is based around learning health systems model, with each cycle and feedback loop issues with data correctness and quality can be discussed and developed. The evolutionary HARMONY Model will improve the systems capabilities and remove errors over time with each cycle. Another strategy for risk mitigation for inaccuracies in data include model personnel and clinical change champions working as guardians of this resource and facilitate transparency, coaching and support within the local contexts.

## 7 CONCLUSION

Looking to the future of healthcare and research there is growing awareness of the importance of implementation science, behaviour change practices and, community and consumer engagement in the utilisation of clinical quality registries to create a learning health system. The HARMONY Model provides a prototype to help reduce clinical variation, enhance research translation capacity, and improve care quality through a transformative approach to health‐service delivery. Using an efficient digital infrastructure as the base allows for rapid implementation of learnings into clinical practice. Presenting an adaptable format that can be implemented within various healthcare settings and catered towards specific health conditions. Ultimately placing the patient, their current health status and their needs at the centre of care provision.

## FUNDING INFORMATION

Kirsten Parker's PhD is supported through an Australian Government Research Training Program (AGRTP) Scholarship. This project is partially funded through a Bequest through the Estate of the late Frank Harvey Smith provided to Professor Richard I Lindley. Professor Caleb Ferguson is supported by a National Health & Medical Research Council Investigator Grant (2020‐2025) Reference—NT1196262. We acknowledge support from Western Sydney Local Health District, Research & Education Network. The funders have no influence on the study conceptualisation, design, discussion or the decision to submit reports for publication.

## CONFLICT OF INTEREST STATEMENT

The authors declare that they have no conflicts of interests.
